# Supplementary material for: A network dynamical simulation model for the study of antibiotic resistance in microbial communities
Source: Gut Microbes. 2026 Sep 19;18(1):2734703. doi: 10.1080/19490976.2026.2734703 (PMC13596919; doi:10.1080/19490976.2026.2734703)
Supplement: Supplementary file 1 [file KGMI_A_2734703_SM9053.zip › supplementary_file_1/Figures/ba_pct_delta_n_edges_n1000.pdf]

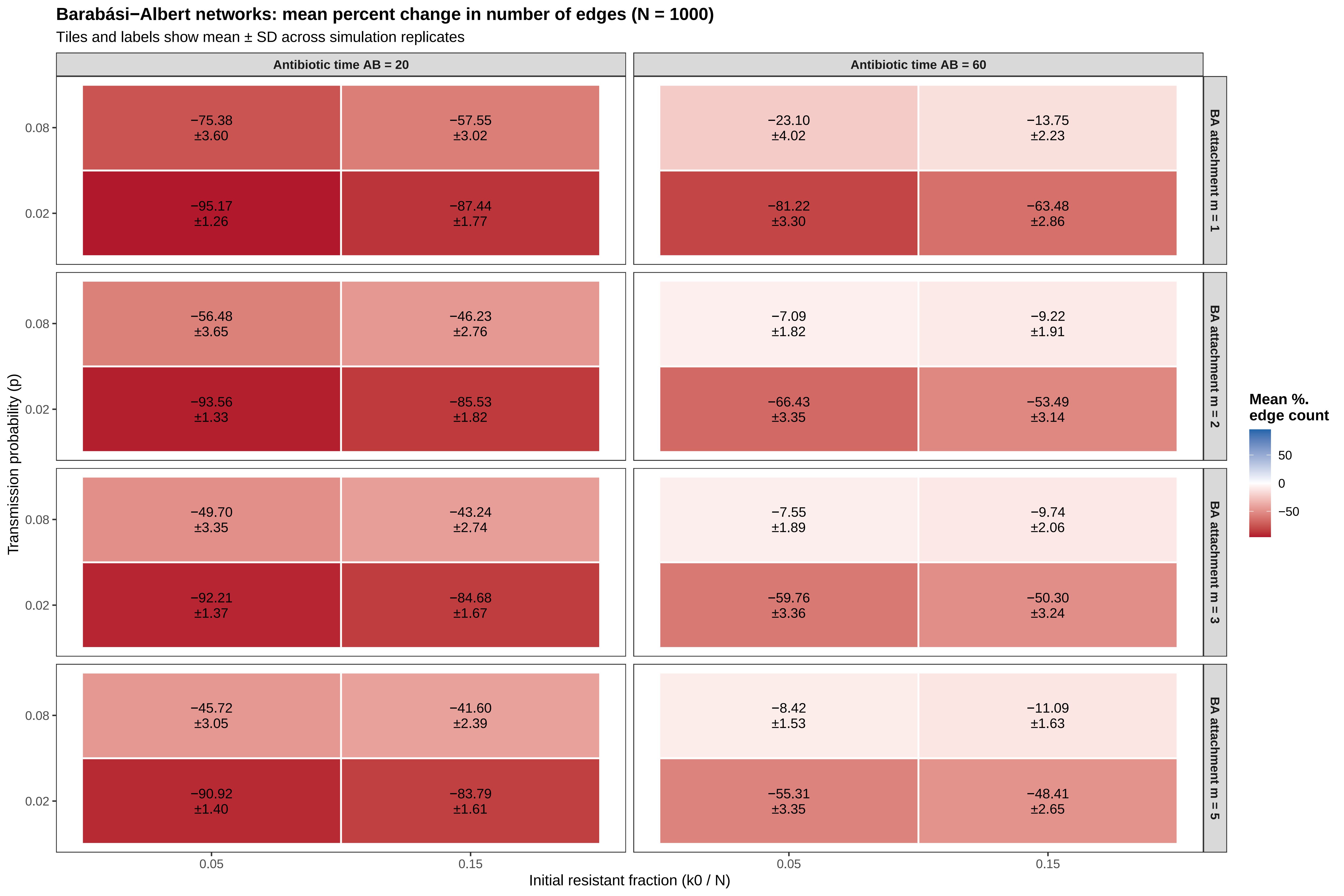
Barabási−Albert networks: mean percent change in number of edges (N = 1000)
Tiles and labels show mean ± SD across simulation replicates
Antibiotic time AB = 20
Antibiotic time AB = 60
BA attachment m = 1
BA attachment m = 2
BA attachment m = 3
BA attachment m = 5
Transmission probability (p)
Initial resistant fraction (k0 / N)
0.08
0.02
0.05
0.15
−75.38 ±3.60
−57.55 ±3.02
−95.17 ±1.26
−87.44 ±1.77
−23.10 ±4.02
−13.75 ±2.23
−81.22 ±3.30
−63.48 ±2.86
−56.48 ±3.65
−46.23 ±2.76
−93.56 ±1.33
−85.53 ±1.82
−7.09 ±1.82
−9.22 ±1.91
−66.43 ±3.35
−53.49 ±3.14
−49.70 ±3.35
−43.24 ±2.74
−92.21 ±1.37
−84.68 ±1.67
−7.55 ±1.89
−9.74 ±2.06
−59.76 ±3.36
−50.30 ±3.24
−45.72 ±3.05
−41.60 ±2.39
−90.92 ±1.40
−83.79 ±1.61
−8.42 ±1.53
−11.09 ±1.63
−55.31 ±3.35
−48.41 ±2.65
Mean %. edge count
50
0
−50
